# On the Dynamics of Active Aging

**DOI:** 10.1155/2012/818564

**Published:** 2012-09-03

**Authors:** Johannes J. F. Schroots

**Affiliations:** ERGO/European Research Institute on Health and Aging; Department of Psychology, University of Groningen, 9712 TS Groningen, The Netherlands

## Abstract

The conceptual basis of active aging is extended with a dynamic systems model, called Janus. The Janus model accounts for the life-course dynamics of simple and more complex growth and decline functions, on the strength of three principles. The first principle of transition states that the unitary lifespan trajectory of development and aging is the product of two complementary forces, growth and senescence, which are effective from conception until death. The first principle solves the traditional problem of the age at which development ends and the process of aging starts. The second and third principles of peak capacity and peak time refer, respectively, to the impact of growth rate (peak capacity) and rate of senescence (peak time) on the life-course of dynamic systems. The validity of the Janus model is demonstrated by simulating the empirical lifespan trajectories of functional capacity, intelligence, and mortality. The Janus model contributes to the concept of active aging by underlining the dynamic limits of human nature, by stimulating effective policies for promoting active aging in the first half of life, and by emphasizing the growth potential of older people in the second half.

## 1. Introduction

The roots of gerontology as a science lie in European scientific developments of the 19th century. The European “Zeitgeist” fostered a strong conviction that the scientific method could be applied to all phenomena and that rational and logical explanations of their causes could be ascertained. Science and its methodology became the doorway to knowledge. In 1835, the Flemish scientist Adolphe Quetelet published the first research report on human development and aging, titled “*A Treatise on Man and the Development of his Faculties”* [1]. The data he reported covered such topics as birth rate, mortality trends by age, stature, weight, and strength, as well as the development of “moral and intellectual qualities of man.” The first sentence of the report reflects his scientific orientation: “Man is born, grows up, and dies, according to certain laws which have never been properly investigated, either as a whole or in the mode of their mutual reactions” (page 1). Next, Quetelet describes an extensive research program for the study of the human life course: 

“… they (i.e.,* Quetelet's colleagues*) have neglected to put forward (…) the study of his physical *development* (bodily growth), and they have neglected to mark by numbers how individual man increases with respect to weight and height—how, in short, his forces are developed, the sensibility of his organs, and his other physical (*and mental*) faculties. They have not determined the age at which his faculties reach their *maximum *or highest energy, nor the time when they commence to *decline*.” (page 1) (italics added JS)

Currently, Quetelet's conception of the life-course is known as a sequential two-phase model of growth and decline, with emphasis on the “developmental” aspects of individual life [2].

One decade before Quetelet, the English actuary Benjamin Gompertz [3] had emphasized the “aging” aspects in a paper “*On the Nature of the Function Expressive of the Law of Human Mortality*.” He made the observation based on death and population records for people in England, Sweden, and France that there is an exponential rise in death rates between ages 20 and 60, that is, the so-called law of mortality. Partly due to the work of Makeham [4], the age range was extended from 10 to 80 years [5].

In the 20th century, Gompertz' name was commonly attached to the full mortality curve for population data, from birth to death. It is important to note that this curve refers not only to the 20–60 or 10–80 age range, but also to the first age period of decreasing mortality from 0 to 10 or 20 years. In other words, the Gompertz curve is characterized by two sequential phases of decreasing and increasing mortality, commonly interpreted as development and aging (senescing), with the minimum varying from 10 to 20 or even 30 years. As such, the Gompertz curve might be conceived as the inverse of Quetelet's growth and decline curve. Both curves confirm the traditional view that the human life course consists of two sequential processes of change, development and aging, with the maximum (Quetelet) or minimum (Gompertz) at maturity or adulthood. This view raises the long-standing, but increasingly relevant question of how development and aging are related [6].

## 2. The Study of the Aged, Age, and Aging

As Gompertz, Quetelet, and many others after them have argued convincingly, aging does not start at 65, and development does not stop at 10 or 20. Nevertheless, both developmental science and gerontology have acted for a long time “as if” they did. Around the turn of the 19th century, one seems to have adapted the concepts of development and aging from areas of disciplines that were not oriented to explaining change. The earlies, biology, psychology, and sociology were based on static properties. This focus gave little encouragement to the study of the dynamics of development and aging. With regard to the field of gerontology, three foci can be distinguished: the aged, age, and aging. 

First, *the study of the aged*—whether the young-old or the old-old—focuses on the characteristics of problematic and nonproblematic elderly while following a thematic approach. Even a cursory glance at the literature shows that there are at least thirty different themes, varying from sensation and perception, attachment and bonding, sexual behavior and personality to depression and dementia, competence and wisdom, age, and the search for meaning [7]. Thematic studies with regard to special services for the elderly (e.g., nursing homes and old people's homes) are not taken into account, but even without these studies the field of gerontology does not provide a coherent sight. Careful descriptions of aged persons are useful as a basis for meeting the needs of the elderly but do not of themselves provide insight into the origins of their needs. For lack of theory, the field has heavily borrowed from older, and certainly more respectable “islands of knowledge” in medicine and psychology. As such, the study of the aged bears a resemblance to the much older field of child medicine and child psychology, which study the first stages of the human life span.

Second, the content of *the study of age* has been largely derived from cross-sectional research, describing and comparing persons of different ages measured at the same time. This approach results in cross-sectional age differences, which are often erroneously interpreted as age changes, aging, or changes in health and behavior over time. It is a persistent fallacy to suggest that some decline on the basis of cross-sectional data is caused by age or the aging process. Although age is a convenient broad index, it is a dummy variable in the sense that it does not stand for a single process but for many processes that may operate independently. The observed decline on the basis of cross-sectional age differences might have been caused by other conditions besides changes with age, like the lower education of the older age group as compared with the higher education of the younger group.

Third, *the study of aging* is concerned with patterns and processes of change in individuals as they develop and grow old. Findings suggest that there is a wide range of individual differences in the rate and manner of aging at all levels of analysis, biological, psychological, and social (cf. *The handbooks of aging* [8], consisting of three volumes, seven editions, 1977–2011). The chronic lack of theory makes it difficult to integrate the different findings. As the classic dictum says “The study of aging is data rich and theory poor, a vast collection of unintegrated pieces of information.” No wonder that so many concepts of aging have been developed, each with a different name and connotation. Negative concepts vary from problematic and abnormal aging to pathological aging, neutral concepts include primary, nonproblematic and normal aging, while positive aging or “aging well” might be referred to as healthy, succesful, optimal or productive aging. All these terms are considered as multidisciplinary, multidimensional, and multilevel concepts. 

In 2002 the WHO has added the new term of *active aging* to the list of positive concepts [9]. Fernandez-Ballesteros [10] summarizes the three most important bases of active aging: (1) compression of morbidity and mortality, (2) the diversity of the ways of aging, and (3) the plasticity of human nature. In this article the basis of active aging will be extended with (4) life-course dynamics [11]. 

## 3. Human Ontogenesis

Birren and Cunningham [12] present a minimal definition of aging as “some typicalness of change over time in mature organisms.” Minimal though it is, the definition nevertheless raises two questions: (a) to what extent are processes of development and aging different from each other, as both processes alike refer to age-related changes, and (b) how is the transition from developmental processes into aging processes to be explained, as aging starts at maturity. The answer to these questions has been given from different theoretical perspectives.

In 1960, Birren presented a general theory of aging as a *counterpart* of development [13]. The use of the metaphor “counterpart” is meant to express the idea that there are latent structures of behavior (emotions, cognitions, and motivations) carried forward from earlier experience that interact with present situations. Aging is viewed as a transformation of the biological and behavioral development of the organism expressed in a “counterpart manner” in variable ecological contexts.

**Figure 1:**
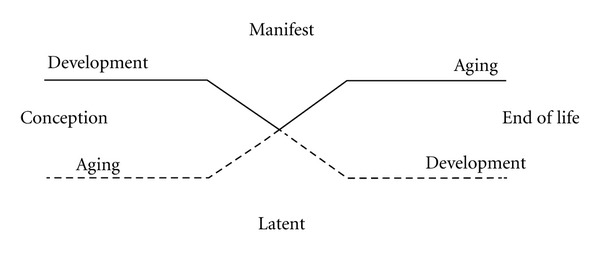
“Butterfly” diagram illustrating the relationship of the processes of development and aging over the course of life [16].

Counterpart theory explains the diachronic relationship between development and aging but does not address the issue of their synchronic relations. To solve this issue Schroots and Birren [14, 15] developed a simple diagram of human ontogenesis, in which development and aging are conceptualized *metaphorically* as two parallel but related processes of change, or as the two sides of a unitary life trajectory (Figure 1). At the start of ontogenesis (conception), the developmental process is the most visible or manifest, while the signs of aging are at the time still obscure or latent, and vice versa at the end of life. It should be noted that across the lifespan, the transition point varies from function to function, from system to system, and from individual to individual. The “butterfly” diagram illustrates clearly the modern conception of development and aging as two simultaneous processes of change, from conception to death, which manifest themselves successively in the form of a unitary life trajectory (solid line). As such, the butterfly diagram functions as a metaphor for a dynamic life-course model, in which aging is the diachronic and synchronic counterpart of development both before and after the transition point [16].

In 1987, Baltes [17] addressed a similar question with regard to the relationship between development and aging, when he introduced the so-called *gain/loss* view of development: 

“According to this view, development at all points of the life course is a joint expression of features of growth (gain) and decline (loss). It is assumed that any developmental progression displays at the same time new adaptive capacity as well as the loss of previously existing capacity. No developmental change during the life course is pure gain” (page 616).

In other words, there is no free lunch in life. By way of illustration, Baltes developed a possible life-span scenario of the dynamics of gains and losses, in which the sum total of possible gains and losses in adaptive capacity shifts proportionally with increasing age [17]. 

**Figure 2:**
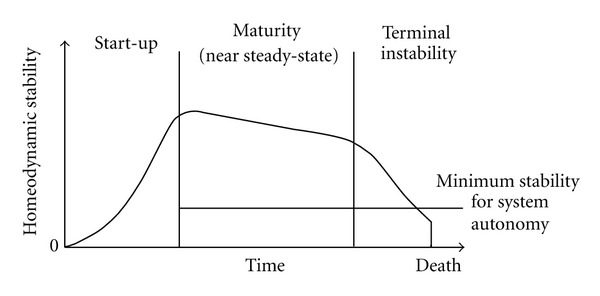
Homeodynamic stability as a function of time in a self-organizing system [19].

Starting from a living systems perspective, Yates [18] developed a prototheory of the *dynamics *of aging. Briefly summarized, Yates hypothesizes that there is some quality informally called vitality or health that expresses the overall dynamic stability of a living system. That stability is homeodynamic, that is, the mean levels of most of the state variables are closely determined, but the variances around them can be very large. Also, the stability of living organisms is not so strict, that they cannot grow and adapt within their lifetimes. The diagram in Figure 2 shows the degree of homeodynamic stability as a function of time in a self-organizing system [19].

A newborn organism is less stable than it will be at maturity. For the human being, the left vertical line, marking the achievement of maturity and maximal stability, occurs at age approximately 30 years, at which time, the capacity of many physiological processes is at its peak. After age 30, there is a linear decline and loss of “reserve,” even when the data are corrected to eliminate disease [20]. Death from “old age” occurs when the physiological losses beginning at maturity have progressed to the point that the stability crosses the minimum required for system autonomy. Translated in dynamic terms, the life trajectory of the self-organizing system begins with growth, development, and differentiation, all of which are negentropic processes that initially mask the ongoing process of senescence. After maturity is reached, the entropic processes become dominant or manifest, leading to a destruction of order in the organism [21, 22].

**Figure 3:**
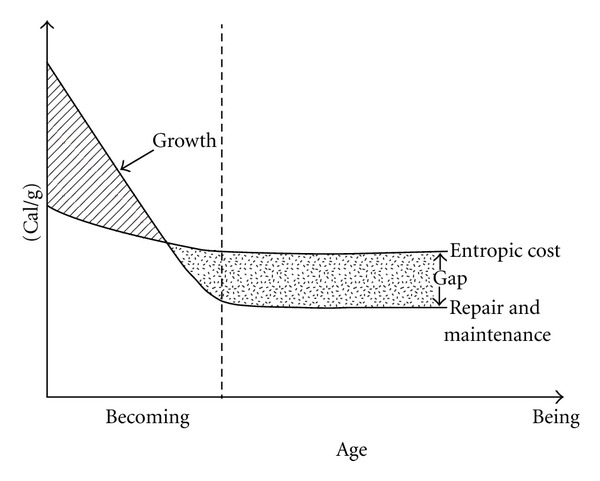
Energetics of a self-organizing system during growth and maturity [22].

In Figure 3, the ordinate shows the energetics (metabolism) of the self-organizing system during growth and maturity. Negentropic (anabolic) processes exceed the entropic (katabolic) costs of repair and maintenance during the stage of growth. After maturity is reached, no net further gain in differentiation occurs and the metabolic process becomes entropic in the net. Figure 3 shows clearly that the process of senescing runs concurrent with growth, development and differentiation in a self-organizing system; but until the growing phase is completed, senescing is masked and not easily detected. In that sense, Yates' homeodynamic perspective on senescing shows some similarity with counterpart theory and gain/loss conceptions of aging, but how the transition occurs from development into aging is not explained.

## 4. Computer Simulation of Development and Aging

**Figure 4:**
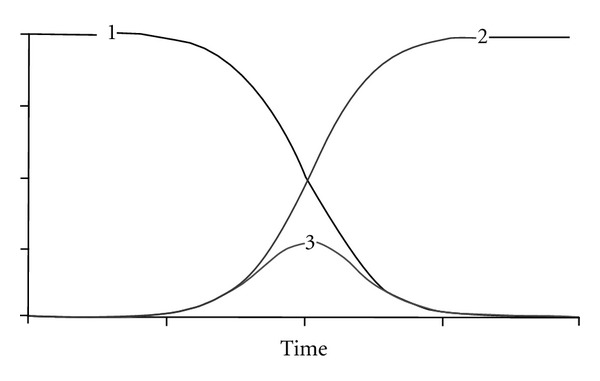
Theoretical model of the “butterfly” diagram: (1) negative growth curve, (2) positive growth curve, and (3) bell-shaped curve [11].

Recently, theoretical progress has been made thanks to the introduction of computer modeling and simulation techniques. The first step in computer simulation relates to the construction of a theoretical model (Figure 4) on the basis of metaphors for development and aging, that is, the “butterfly” diagram (Figure 1).

**Figure 5:**
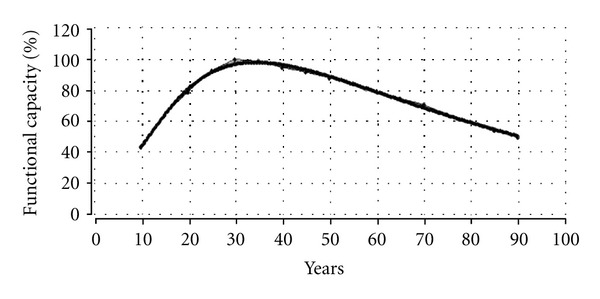
Life trajectory simulation of the functional capacity in human beings [11, 25].

The model in Figure 4 shows three curves: Curves  1 and  2 represent the mathematical equivalent of development (negative growth) and aging (positive growth), respectively, as shown in the “butterfly” diagram of Figure 1; the bell-shaped curve 3 is the mathematical product of development and aging (solid line in Figure 1) and represents the unitary life trajectory of a virtual system with the apex (peak) at the *transition* point of development and aging. The three curves have been made symmetrical to illustrate the similarity to Figure 1. However, the peak of the life trajectory (curve 3) might move up or down (*peak capacity*), and to the left or right (*peak time*), depending on the growth parameters of curves 1 and 2, respectively (e.g., the asymmetrical graph in Figure 5).

It should be noted, firstly, that the representation of development as negative growth seems paradoxical, but is in fact logical, viewed from the central idea that the growth rate is highest at birth (or conception) and steadily declines thereafter [23, 24]. Following the same line of reasoning, aging is conceived, conversely, as the process with the lowest growth rate at birth and the highest rate at the end of life. The second note is that the mathematical equivalents (differential equations) of the three simulated curves are at the basis of a dynamic life-course model, which is called *Janus* after the Roman god with two faces—one face looking into the future and one into the past [11]. The Janus model has simple and more complex versions (see Appendix). Both versions will be used for the next step. 

The second step relates to the simulation of empirical data. By way of illustration, we will simulate the life trajectories of (a) general physiological performance, (b) fluid and crystallized intelligence, and (c) U.S. mortality in the years 1910 and 1970.

**(a) General Physiological Performance**

Starting from various datasets, Kemper and Binkhorst [25] plotted the idealized life trajectory of the functional capacity in human beings. We have simulated this trajectory with the simple version of the Janus model, and the resulting graph (bold line) in Figure 5 seems to fit almost perfectly the empirical data set of functional capacity over a period of 10 to 90 years, with a peak performance of 100% at ca. 30 years and 80% performance at the age of 60.

It should be noted that the combined forces of life, that is, growth and senescence—not shown in Figure 5, but similar to the forces of negative growth (curve 1) and positive growth (curve 2) in Figure 4—influence the life trajectory of development and aging in such a way that the peak (apex) of functional capacity is reached at about 30 years, after which the vitality or functional capacity declines gradually, varying between 0.5 and 1.0% per year depending on the individual and the organ system, but in this case declining with ca. 0.7% per year [20].

The implications of the simple Janus model for the physiological performance of people in general can be demonstrated by means of two well-known sayings *Soon ripe*, *soon rotten*, and *Live fast*, *die young*. Peak capacity, for example, relates to the phenomenon that rapid growth in the phase of development leads to rapid decline (Soon ripe, soon rotten), with the result that the functional capacity of the individual reaches its critical capacity for survival at a younger age than would have been the case with slow growth (on the condition that the rate of senescence is constant). Peak time, on the other hand, is related to rate of senescence: higher rates of senescence (at a constant growth rate) mean that the individual reaches his or her peak and critical threshold at an increasingly early age (Live fast, die young).

**(b) Fluid and Crystallized Intelligence**

Simple life-trajectories show two phases: development and aging, and one transition. Development is often compared with incremental processes of change and aging with decremental processes. The classic metaphor for biological processes of change is that of the *hill *with the top at about 30 years for general physiological performance [16]. For a few decades, however, the notion has been growing that psychological processes of change do not necessarily parallel biological changes along the lifespan. The psychological attribute of wisdom, for instance, represents a progressive aspect of change in middle and late adulthood and challenges the traditional decline view of aging [26]. The question now arises whether there is a fit between the Janus model and, for instance, general intelligence or mental abilities.

**Figure 6:**
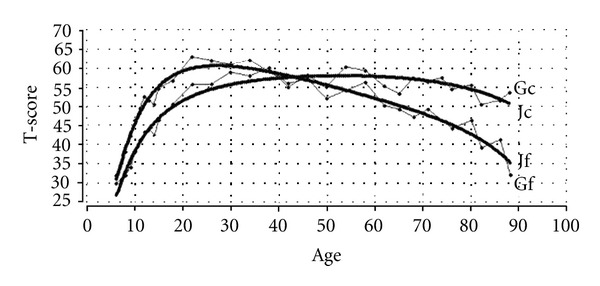
Life trajectory simulation of fluid (Gf) and crystallized (Gc) intelligence (dotted) with the Janus model (bold): Jf = fluid; Jc = crystallized [11, 28].

Traditionally, general intelligence is divided into two types of mental abilities: “fluid” or spatial-analytical abilities (abstract reasoning), which refer to basic processes of speed of information processing, and “crystallized” abilities, which refer to the storage of information (e.g., cultural knowledge and experience). The lifespan patterns of both abilities show a rapid rise until early adulthood, followed by a period of relative stability in respect of the crystallized abilities until the age of about 60–70 years, but a distinct decline in the fluid abilities after early adulthood [27]. As such, the lifespan curve of fluid abilities bears a strong resemblance to the general physiological performance curve (two phases, one transition), while the crystallized abilities curve includes an extra relative stability phase between early adulthoud and the later years, that is, three phases and two transitions. In order to simulate these divergent lifespan patterns, a more complex, but essentially similar version of the Janus model has been used. In Figure 6, the lifespan curves of fluid (Gf) and crystallized (Gc) intelligence are presented (dotted lines, copied from Li et al. [28]), as well as the simulated fluid (Jf) and crystallized (Jc) Janus curves (bold lines), from Schroots [11].

Figure 6 shows a satisfactory fit between the simulated Janus curves and the empirical fluid and crystallized intelligence graphs. As expected, fluid intelligence follows the traditional pattern of development (6–27 yrs) and aging (27–88 yrs) with the peak at about 27 years. Birren and Fisher [29] explain this pattern by noting that fluid abilities are primarily based on the neurobiological property of *information processing speed*. As such, the dynamics of fluid intelligence generally corresponds to the life trajectories of many other biological systems that reach their peak performance in early adulthood and decline afterwards [20] (see Figure 5). The neurobiological roots of fluid intelligence imply that the individual's fluid capacity is as little modifiable as his or her functional capacity. 

In contrast to fluid intelligence, crystallized intelligence continues to develop, though more slowly, from age 27 to the peak age of about 50 years then stabilizes more or less at peak level until the 60–70 age period, thereafter, finally declining more rapidly until the end of life. If fluid intelligence relates to speed of information processing, then crystallized intelligence relates to *storage of information* (memory, knowledge, experience), which is less susceptible to neurobiological decline. Information processing precedes storage of information, which makes it likely that crystallized intelligence is composed of both information processing and storage of information. From a dynamic systems perspective, crystallized intelligence, therefore, rides piggyback on fluid intelligence, which explains crystallized abilities' period of relative stability after early adulthood until their distinct decline at an advanced age. The partly neurobiological, partly cognitive, and cultural roots of crystallized potential imply that specific mental change processes, like life-long accumulation of knowledge, wisdom, and experience, are a reality until late in life.

**(c) Mortality**

**Figure 7:**
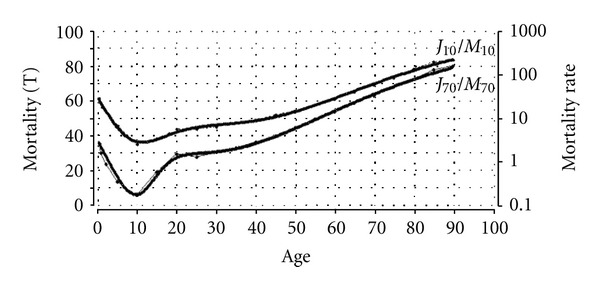
Simulation of U.S. mortality in the years 1910 and 1970 (dotted: *M*_10_, *M*_70_) with the Janus model (bold: *J*_10_, *J*_70_) [11, 30].

The third empirical dataset relates to mortality and is borrowed from Fries and Crapo [30], who presented a graph of vital statistics from the United States for 1910 and 1970. For scale-technical reasons, the semilogarithmic mortality data were transformed linearly before the copied graph data were imported into the simulation program.  Figure 7 presents the empirical mortality data for the United States in the years 1910 (*M*_10_) and 1970 (*M*_70_), as well as the simulated Janus curves *J*_10_ and *J*_70_ [11]. Note that the transformed (T) linear scale of the left *y*-axis (0–100) is labeled “Mortality (T)” and that the original, logarithmic scale of the right *y*-axis (0.1–1000) is labeled “Mortality Rate.”

Figure 7 shows an almost complete overlap between the Janus curves (bold) and the mortality data (dotted). As such, the fit between the Janus curves (*J*_10_, *J*_70_) and data plots (*M*_10_, *M*_70_) is more than satisfactory. Both Janus curves can be characterized in terms of development (decreasing mortality) and aging (increasing mortality), with the lowest probability of death (minimum) at the age of about 10. However, there are also differences between the *J*_10_ and *J*_70_ curves. First, the minimum mortality was much higher in 1910 than in 1970. This huge drop in mortality is generally attributed to the improvement in the overall health of western populations. Second, the Janus curves, particularly the *J*_70_ curve, are not smooth over the ages 10–30 (*J*_70_) and 10–45 (*J*_10_); this irregularity or “bump” in the mortality curve represents traumatic deaths (accidents), which peak during these ages. 

In this context, Fries and Crapo [30] maintained that since 1910 “the relative importance of trauma has increased greatly; such deaths made up nearly 75% of all deaths between ages 15 and 25” (page 28). After the “bump” period, the mortality rate of both curves displays an upward slope until the age of ca. 90.

**Figure 8:**
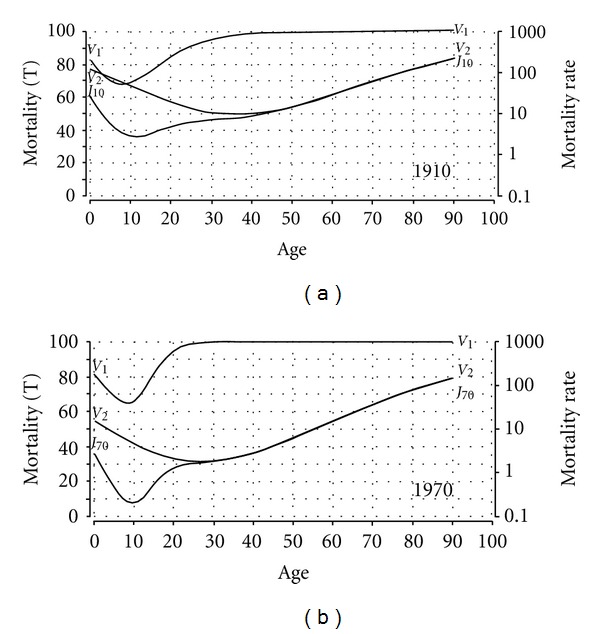
(a): Janus curve (*J*_10_), *V*_1_ and *V*_2_ curves of U.S. 1910 mortality. Bottom panel: Janus curve (*J*_70_), *V*_1_ and *V*_2_ curves of U.S. 1970 mortality.

From an analytical perspective, the Janus curves can be resolved into system curves *V*_1_ and *V*_2_. In Figure 8, the Janus curves, as well as the *V*_1_ and *V*_2_ curves are presented for the years 1910 (top panel) and 1970 (bottom panel). The data plots have been omitted from the figure for convenience and visibility. Note that, due to the limited graphical resolution, the exact bifurcation points of the Janus and system curves only become visible after enlargement of the figure.

Figure 8 indicates that the minimum mortality of both the Janus curves practically coincides with the transition (minimum) of the *V*_1_ curves at the age of ca. 10. After visual inspection of the *V*_2_ curves in the enlarged figure, it was determined that the *V*_2_ minimum for 1970 (bottom panel) coincides with the Janus curve at the end of the “bump” period (ca. 27–30 years), and that the *V*_2_ minimum for 1910 (top panel) is reached at the age of ca. 37. The latter is well before the end of the “bump” period (ca. 45–50 years). The question arises how the first (*V*_1_) and second (*V*_2_) systems in the Janus model framework should be interpreted.

With regard to the first system, the interpretation should not be too difficult, that is, the *V*_1_ curve reflects the impact of environmental and pathological conditions, as well as of accidents on individual lives. The reasoning is as follows. Given negative prenatal, perinatal, and postnatal conditions (e.g., congenital defects, infectious diseases), infant mortality will be high and, consequently, the impact of accidents in adolescence and young adulthood will be relatively low, as shown for the year 1910 in which the “bump” is hardly visible. However, if the overall health conditions are improved, as in 1970 western society with its public services (e.g., potable drinking water, community health programs, sewers, etc.), infant and childhood mortality will be low. Consequently, the “bump” mortality of adolescence and young adulthood emerges relatively distinct from what is presumably the natural, intrinsic mortality of human beings as reflected by the *V*_2_ curve (see bottom panel of Figure 8). In other words, the first system of *extrinsic* mortality dominates the second system of *intrinsic *mortality, but in combination they produce the full Gompertz curve as simulated by the more complex version of the Janus model.

On the whole, the *V*_1_ curve primarily reflects the shift in the mortality dynamics of an increasingly healthy (or unhealthy) population [31]. The *V*_2_ curve, on the other hand, expresses the basic mortality dynamics in the form of an inverse growth and decline function that is intrinsic to the human organism, and relatively independent of the environment. 

## 5. The Janus Model of Life-Course Dynamics

Generally, the Janus model offers a quite satisfactory account of the life-course dynamics of simple and more complex growth and decline functions. The simple version of the Janus model (two phases, one transition) is based on the simultaneous and complementary action of two coupled forces, growth and senescence, which determine the dynamics of living systems, or—to put it differently—define the one-peak life trajectories of dynamic systems. The extended version of the Janus model covers the more complex, two-peak trajectories (three phases, two transitions). Note that the forces (processes) of growth and senescence should be conceived as postulates of the same order as the physical force of gravity, which does not as yet have a clear explanation. 

The term “living system” is extracted from Miller's systems theory [32], which states that humans are primarily regarded as living systems, hierarchically organized from many subsystems such as cells, cell tissues, organs, among others, according to their complexity levels. As a system, humans can be conceived as part of an even more complex, larger system, for example, the social and physical environment. From the latter point of view, it depends on the system level whether the term “living system” or “dynamic system” is used. Whatever term is selected, the Janus model is primarily a mathematical, “empty” model that fits the growth and decline curves of widely divergent systems from biological and psychological systems to social and demographic systems. However, once interpretation and context are added, the mathematical model loses its separate identity, and the scientist finds him or herself in the process of theory development. 

The construction of the Janus model revealed three principles. The first principle of *transition* solved the traditional problem of the age at which development ends and the process of aging starts. This principle states that the apparent unitary lifespan trajectory of development and aging is in fact the product of two complementary forces, growth and senescence, which are effective from conception until death. The second and third principles of *peak capacity* and *peak time* refer, respectively, to the impact of growth rate (peak capacity) and rate of senescence (peak time) on the life-course of dynamic systems and of human beings in particular. Different growth rates with a constant rate of senescence have implications for the peak capacity and the residual lifespan after the transition point. Rapid growth, for example, leads to a higher peak at a certain age, and also to rapid decline and a shorter residual lifespan than slow growth, which results in a lower peak, slower decline, and a longer residual life trajectory after the point of transition. On the other hand, different rates of senescence with a constant growth rate mainly have implications for peak time (age), peak capacity, and the total lifespan. Rapid senescence, for example, results in a higher peak at a younger age, and also in a shorter lifespan than slow senescence with a lower peak at an older age and a longer total lifespan. 

Generally, growth rate refers to the system's maximum capacity (2nd principle) and the rate of senescence refers to the age at which the system reaches its maximum capacity (3rd principle). Note, however, that the forces of growth and senescence are confounded, unless one of the two forces is kept constant, for example, in a quasi-experimental design.

## 6. Active Aging from a Dynamic Perspective

The question arises what the Janus model might contribute to the life-course dynamics of active aging. Starting from Fernandez-Ballesteros' summary [10] of the three bases of active aging, the first base with respect to the *compression of morbidity and mortality* will be illustrated with the simulation of U.S. mortality for 1910 and 1970 (see Figure 8). It was found that the Janus model for two coupled systems could resolve overall mortality in two components, tentatively labeled as intrinsic and extrinsic mortality. The first component of extrinsic mortality (1st system) reflects the fatal impact of environmental and pathological conditions, as well as accidents on individual lives. The extrinsic mortality curve indicates a minimum in respect of the age of ca. 10 and extends from birth to about the age of 50 in 1910 and to about age 30 with regard to the 1970 data. According to the third principle of peak time, this means that the rate of senescence in the first system is constant for both 1910 and 1970. The second principle of peak capacity (growth rate) thus explains the differences between the first system's 1910 and 1970 curves with regard to the minimum and residual trajectories. In other words, the rapid decrease of negative conditions in 1970 leads to a lower minimum mortality and shorter residual trajectory (ca. 10–30 yrs) than the slow decrease of 1910 with its higher minimum mortality and longer residual trajectory (ca. 10–50 yrs). From this perspective, the 1910 and 1970 extrinsic mortality curves serve as example of a quasi-experimental, demographic design with a variable peak capacity and constant peak time. 

The second component of intrinsic mortality reflects the human organism's inherent, natural capacity to adapt to life. The intrinsic mortality curve (2nd system) extends over the full lifespan (0–90 yrs) for both 1910 and 1970 with minimum mortality from about age 37 in 1910 to age 27 in 1970. Note that the age shift of minimum intrinsic mortality from 37 to 27 years is coupled with an improvement in living conditions and lower extrinsic mortality. Note, moreover, that the minimum intrinsic mortality in 1970 (ca. 27 yrs) corresponds to the peak of general physiological performance at about the age of 30 [25]. It is, therefore, not unreasonable to assume that intrinsic mortality's inverse growth and decline curve reflect some basic survival mechanism, also called adaptability or functional fitness, which reaches its maximum strength at the age of ca. 30. 

Generally, the compression of morbidity and mortality is reflected in the shift over time and/or level of extrinsic and intrinsic mortality. The environment's impact on mortality (*nurture*) dominates in the first 30 to 50 years of life and from then onwards our mortal *nature* becomes manifest. It would be interesting to learn whether the Janus model of two coupled systems could clarify other nature-nurture problems as well. 

**Figure 9:**
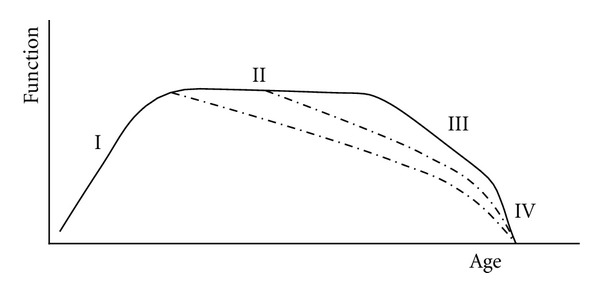
Idealized life trajectories of functional performance. Some functions (- - - -) decline earlier than others [33].

The second base of active aging refers to the *diversity of the ways of aging*. Varied life trajectories reflect among other things the impact of general aging processes on the course of life. The diverse trajectories have been demonstrated by means of the Janus model at the psychological (intelligence) and demographic (mortality) levels of dynamic systems (Figures 6, 7 and 8), but not at the biological (health) level. Starting from the Gothenburg longitudinal dataset, Svanborg [33] illustrated the dynamics of aging at the functional performance level in the form of a diagram showing four phases (Figure 9):

“ … the first illustrating *growing and maturation*, the second a commonly occurring more or less *stationary phase*, a third with a decline in *functional performance*, usually about 1% per year, and finally a *terminal phase* with an accelerated rate of aging (…). It might be reasonable to predict that during this terminal phase with a rapid decline in functional performance the reserve capacity of different organs and organ functions should be very low, and that possibilities for stimulation of, for example, muscle function during that period might be very limited or nonexistent.” (page 137). 

The diagram in Figure 9 represents the idealized composite of three datasets, which illustrate both the simple (1-peak) and complex (2-peak) Janus model of life-course dynamics. The first (I) phase of growing and maturation, followed by the first dotted trajectory of decline, corresponds with the simple Janus model of development and aging (cf. Figure 5). The complex, 2-peak Janus model might cover the first (I), second (II), and third (III) phases of the total life trajectory, that is, development (growth)—stationary phase—aging (decline), and could also be fitted to the last three phases (II, III, IV), depending on the model parameters (cf. Figure 6). Note that the diagram in Figure 9 shows only a few of all possible aging trajectories. It would be interesting, therefore, to find some order in the confusing diversity of aging processes.

**Figure 10:**
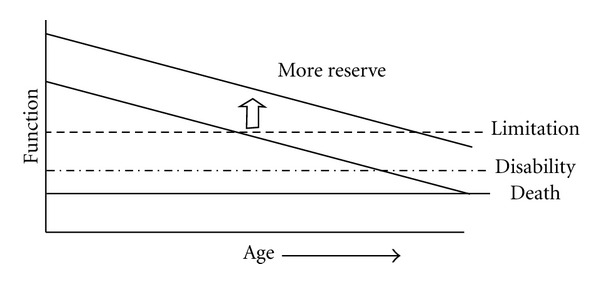
Hypothesized relationship between physiologic aging, functional health and chronological age [34].

Figure 10 shows a diagram of the hypothesized relationship between physiologic aging, functional health and chronological age, as presented by Kritchevsky [34]. The diagram illustrates how the three Janus principles of transition, peak capacity, and peak time might be applied in disentangling the underlying mechanisms of diverse aging trajectories. 

First, note that the developmental trajectories are not shown in the diagram for better understanding of the parallel aging trajectories. Next, it is assumed that the *y*-axis of functional health represents the transition between development and aging. This means—according to the simple 1-peak Janus model—that the peak time of aging trajectories is constant and that their peak capacity varies, depending on the rate of development. Given the higher and lower peak capacity of the two parallel aging trajectories, the diagram shows clearly the late life health effects (limitation, disability, death) of both rate of development and differences in reserve capacity. In case of nonparallel trajectories (not shown), the complex 2-peak Janus model should be applied. 

The third base of active aging refers to the *plasticity of human nature*. “Plasticity” is the protean term for saying that human beings can change over the course of life and that specific changes like active aging can be effected by policies and interventions. Obviously, unlimited change is out of the question. A fruitful approach would be, therefore, to emphasize the dynamic limits of human nature in the first place, rather than its plasticity. For instance, since the beginning of the last century, about 30 years have been added to the average life expectancy, at least in developed countries. Against this rapid increase in length of life, life-course dynamics sets a probabilistic maximum life-span potential of 120 to 130 years with concomitant rectangularization of the survival curve [19, 22]. Given the natural limits, simulation of the mortality curve with the Janus model would predict increasing rectangularization, as a result from increased survival at all ages, but especially at the young and middle ages [11]. Consequently, effective policies for promoting active aging should be considered primarily in the first 30 to 50 years of life rather than in the second half [35].

Vital statistics like mortality and morbidity have a negative connotation, due to the emphasis on the limits of human nature. On second thoughts, a positive approach of active aging would focus on its dynamics and use the term *human potential* within the natural constraints of life. In the *Mature mind*, Cohen [36] presents an overview of what happens to the brain as it ages, and what effects those changes have on our lives: (a) the brain has the capacity to “remodel” itself—certain genes are activated by experience as we age, allowing our personalities to grow and change in surprising ways (b) the brain can “recruit” areas of itself that were formerly underused, thus these strength and agility reserves can compensate the aging effects in other parts of the brain and (c) it is in the latter stages of life, at age 60–80, that the brain's “information processing centre” achieves its greatest density and reach. In other words, Cohen emphasizes the hidden growth potential of individuals in the second half of life.

In 2008, Becker and Schroots [37] published a volume, titled *Releasing the potentials of senior scholars and scientists*, a.o. composed of the following chapters: “The hidden resources and life-course dynamics of academics, particularly the lifespan development of their physical and mental abilities” (Schroots); “The consequences of the pattern of generations in science” (Becker); “The negative effects of mandatory retirement on active aging and the availability of scientific personnel” (Fernandez-Ballesteros et al.); “Senior scholars and scientists' various role models” (Birren et al.). In these chapters, the contours of a new research domain become visible, in which the *growth potential* of older people is explored after the transition point of development and aging [38, 39]. Expectations are that the Janus model of life-course dynamics can play an important role in the further development of the field.

## 7. Summary

In this article, the dynamics of active aging is discussed at different levels of theorizing, varying from the level of history and metaphor to the level of model and theory. Starting from the early history of development (Quetelet) and aging (Gompertz), a brief overview is presented of the study of the aged, age and aging. Integration of the different findings turns out to be difficult, due to a chronic lack of theory.

At the level of metaphor, three conceptions are discussed: counterpart (Birren), butterfly (Schroots and Birren), and gain/loss (Baltes). The counterpart metaphor explains the diachronic relationship between processes of development and processes of aging, in addition to which the butterfly metaphor assumes a synchronic relationship from conception to death. The gain/loss view of development implies that development at all points of the life course is a joint expression of features of growth (gain) and decline (loss). 

From a theoretical perspective, the discussion is focused on the Dynamics of self-organizing systems (Yates and Schroots). Development and aging, defined as primarily negentropic and entropic processes, respectively, are conceptualized as two parallel but related processes of ontogenetic change or as two sides of a unitary life trajectory.

On the basis of the “butterfly” metaphor for development and aging, a dynamic systems model is presented, that is, the Janus model (Schroots), which offers a quite satisfactory account of the life-course dynamics of simple and more complex growth and decline functions. The Janus model is characterized by three principles: transition, peak capacity, and peak time. The first principle of transition solves the traditional problem of the age at which development ends and the process of aging starts. This principle states that the apparent unitary lifespan trajectory of development and aging is the product of two complementary forces, growth, and senescence, which are effective from conception until death. The second and third principles of peak capacity and peak time refer respectively to the impact of growth rate (peak capacity) and rate of senescence (peak time) on the life course of dynamic systems. The validity of the Janus model is demonstrated by simulating the empirical lifespan trajectories of functional capacity, intelligence, and mortality.

In conclusion of the article, the contribution of the Janus model is discussed in respect of the life-course dynamics of active aging. Starting from the three bases of active aging—compression of morbidity and mortality, diversity and plasticity—the Janus model contributes to the concept of active aging by underlining the dynamic limits of human nature, by stimulating effective policies for promoting active aging in the first half of life, and by emphasizing the growth potential of older people in the second half. The Janus model of life-course dynamics can play an important role in establishing the fourth base of active aging. 

## Appendix

Simple Janus Model Processes of development and aging are reduced to the more elementary form of one-dimensional growth, which follows an S-shaped power curve in which there is a limit to growth, that is, the logistic or limited growth curve. Mathematically, the logistic curve can be expressed in a differential equation in terms of either negative or positive growth, where the limit *C* is constant. Development and aging are then reduced to, respectively, negative (*x*) and positive (*y*) growth:

(A.1) $$\begin{array}{c} \frac{dx}{dt}=-\frac{x\;\left(C-x\right)}{C}, \end{array}$$

(A.2) $$\begin{array}{c} \frac{dy}{dt}=\frac{y\;\left(C-y\right)}{C}. \end{array}$$

Starting from Herbart's formula [40]: *xy*/(*x* + *y*) or *xy*/*C*, equations (A.1) and (A.2) can be rewritten in terms of variable *V* (=living system). Mathematical coupling of the two equations results in differential equation (A.3), which describes the simple life trajectory of a living system:

(A.3) $$\begin{array}{c} \frac{dV}{dt}=\frac{V\left(x-y\right)}{x+y}=\left[\frac{x-y}{C}\right]V, \end{array}$$

Complex Janus Model Differential equation (A.3) of the simple model can be extended with an extra mathematical term that is equal to the original equation. Mathematical coupling of the two differential equations will then result in differential equation (A.4) of what has been called the more complex *Janus* (*J*) model after the Roman god with two faces—one face looking into the future and one into the past Schroots [11]:

(A.4) $$\begin{array}{c} \frac{dJ_{V}}{dt}=\frac{V_{1}\left(x-y\right)}{x+y}+\frac{V_{2}\;\left(v-w\right)}{v+w}. \end{array}$$

Analogous to Herbart's formula, (A.5) shows in a contracted form the formula of the more complex Janus model (*J*_*V*_) for two living systems (*V*_1_, *V*_2_):

(A.5) $$\begin{array}{c} J_{V}\;=\;V_{1}\;+\;V_{2} \end{array}$$

in which *V*_1_ = *pxqy*/(*px* + *qy*) and *V*_2_ = *rvsw*/(*rv* + *sw*), parameters *p* and *r* represent the rate of growth, and parameters *q* and *s* the rate of senescence.
